# The Diagnostic Potential of RNA Aptamers against the NS1 Protein of Dengue Virus Serotype 2

**DOI:** 10.3390/biology12050722

**Published:** 2023-05-15

**Authors:** Ramesh Thevendran, Sivalingam Rogini, Glenn Leighton, Atherton Mutombwera, Sarah Shigdar, Thean-Hock Tang, Marimuthu Citartan

**Affiliations:** 1 Department of Biomedical Science, Advanced Medical & Dental Institute (AMDI), University Sains Malaysia, Bertam, Kepala Batas 13200, Malaysiatangth@usm.my (T.-H.T.); 2 Hutano Diagnostics Ltd. BioEscalator, Innovation Building, Old Road Campus, University of Oxford, Roosevelt Drive, Oxford OX3 7FZ, UK; glenn@hutano-diagnostics.com (G.L.);; 3 School of Medicine, Deakin University, Geelong, VIC 3217, Australia; 4 Institute for Mental and Physical Health and Clinical Translation, School of Medicine, Deakin University, Geelong, VIC 3220, Australia

**Keywords:** RNA aptamers, truncated, aptamer-based ELASA assay

## Abstract

**Simple Summary:**

Dengue infection, a global threat, necessitates prompt action. An early diagnosis based on the dengue antigenic target is preferable. The current method of dengue diagnosis is based on antibodies, which have many disadvantages such as expensive production process and non-uniform batch synthesis. Aptamers are able to address shortcomings of antibodies, and with this in mind, we set out to isolate RNA aptamers against the NS1 protein of dengue virus serotype 2. In this study, we isolated two aptamers known as DENV-3 and DENV-6. Upon truncation to TDENV-3 and TDENV-6a, their performance in the direct ELASA improved. Using these aptamers, a sandwich ELASA was successfully developed for the detection of dengue NS1. The sensitivity of the sandwich ELASA was further improved by stabilization of the truncated aptamers and our repeated incubation strategy, attaining a LOD of 2 nM using NS1 spiked into human serum prepared using a dilution factor of 1:2000.

**Abstract:**

Dengue infection, caused by the dengue virus, is a global threat which requires immediate attention and appropriate disease management. The current diagnosis of dengue infection is largely based on viral isolation, RT-PCR and serology-based detection, which are time-consuming and expensive, and require trained personnel. For early diagnosis of dengue, the direct detection of a dengue antigenic target is efficacious, and one such target is NS1. NS1-based detection is primarily antibody-centric and is beset by drawbacks pertaining to antibodies such as the high cost of synthesis and large batch-to-batch variation. Aptamers are potential surrogates of antibodies and are much cheaper, without exhibiting batch-to-batch variation. Given these advantages, we sought to isolate RNA aptamers against the NS1 protein of dengue virus serotype 2. A total of 11 cycles of SELEX were carried out, resulting in two potent aptamers, DENV-3 and DENV-6, with dissociation constant values estimated at 37.57 ± 10.34 nM and 41.40 ± 9.29 nM, respectively. These aptamers can be further miniaturized to TDENV-3 and TDENV-6a with an increased LOD upon their usage in direct ELASA. Moreover, these truncated aptamers are highly specific against the dengue NS1 while showing no cross-reactivity against the NS1 of the Zika virus, the E2 protein of the Chikungunya virus or the LipL32 protein of Leptospira, with target selectivity retained even in human serum. The usage of TDENV-3 as the capturing probe and TDENV-6a as the detection probe underpinned the development of an aptamer-based sandwich ELASA for the detection of dengue NS1. The sensitivity of the sandwich ELASA was further improved with the stabilization of the truncated aptamers and the repeated incubation strategy, which enabled a LOD of 2 nM when used with the target NS1 spiked in human serum diluted at 1:2000.

## 1. Introduction

Caused by dengue virus, dengue viral infection or dengue fever is one of the most important and widely studied vector-borne diseases. Dengue infection is more prevalent in Asian countries, contributing to 70% of the global burden [1]. Specifically, ASEAN countries have reported an increase of 46% in dengue cases from 2015 to 2019, with Indonesia, Myanmar and Thailand being the epicentre of dengue infection [2]. The most challenging aspect of dengue infection management is during the acute phase, whereby rapid and precise diagnosis is required for better disease management. Viral isolation and identification are tedious and time-consuming, taking as much as 7 to 14 days. Viral RNA detection via RT-PCR is laborious, expensive, and prone to false positivity. Serological testing based on IgG and IgM is beleaguered by the lengthy time necessary for the level of antibodies to rise to a detectable level. Therefore, serology-based detection is unsuitable for early detection. Detectable in the acute-phase sera of infected individuals as early as the first day after the onset of fever [3], the dengue NS1 protein is the most appropriate biomarker for early dengue diagnosis. In fact, among the seven non-structural proteins (NS1, NS2a, NS2b, NS3, NS4a, NS4b and NS5), NS1 is considered the main driving factor for the pathogenesis of the dengue virus. A strong correlation is observed between the plasma/serum levels of secreted NS1 protein and the severity of the viral infection in clinical patient samples. Moreover, patients with elevated NS1 plasma concentrations are indicative of more rapid disease progression to dengue haemorrhagic fever (DHF) compared to patients with mild dengue fever and lower NS1 levels [4]. Similarly, greater levels of secreted NS1 protein are also detected in patients with secondary infections of other dengue serotypes following a primary infection [3]. In the bloodstream of DHF/DSS patients, the level of NS1 protein could reach as much as 50 µg/mL [3]. Collectively, these features associated with NS1 protein clearly signify the promising diagnostic value of the biomarker.

The current diagnostic strategies used for the detection of NS1 are based on the usage of antibodies [5]. Antibodies suffer from several disadvantages such as batch-to-batch variation, inherent irreversibility, expensive and temperature instability. To compensate for these shortcomings, a superior molecular recognition element is required in order to create a more robust diagnostic platform. Aptamers, single-stranded nucleic acids that have excellent specificity against a plethora of target molecules, are the potential surrogate of antibodies. Compared to antibodies, aptamers show no batch-to-batch variation, have lower cost of production and possess reversible denaturation properties as well as being stable at room temperature for long-term storage. The advantages of aptamers relative to antibodies enable them to serve as the prolific molecular recognition element in various diagnostic platforms such as electrochemical-based sensors, histostaining applications and lateral flow assay [6]. The disadvantages associated with antibodies motivate the effort to shift from antibody-centric diagnostics to aptamer-based diagnostics of the NS1 protein.

Several authors have successfully deployed aptamers in detecting viral antigenic targets. Lee et al. developed an aptamer-based ELISA platform for the detection of NS1 proteins of the Zika virus [7]. The system demonstrated high specificity and the limit of detection (LOD) of 0.1 pg/mL. Zou et al. also utilized aptamers in detecting the capsid protein VP1 of enterovirus EV-A71, with a LOD of 200 ng/mL [8]. Similarly, Chen et al. also utilized an aptamer-based ELISA system in detecting the SARS-CoV-2 nucleocapsid protein, exhibiting a LOD of 10 ng/mL [9]. In this study, we sought to isolate RNA aptamers against the target NS1 of dengue virus serotype 2 and investigate their diagnostic potentialities. Two aptamers, DENV-3 and DENV-6, were isolated with the dissociation constant values estimated at 37.57 ± 10.34 nM and 41.40 ± 9.29 nM, respectively. Upon truncation, these two aptamers showed improved binding to the target dengue NS1, while showing no cross-reactivity to the NS1 protein of Zika virus, the E2 protein of Chikungunya virus and the LipL32 protein of Leptospira, and they retained high selectivity in human serum. Using these two aptamers, an aptamer-based sandwich ELASA assay was developed with the LOD of 1 nM achieved in human serum diluted at a dilution factor of 1:2000.

## 2. Materials and Methods

### 2.1. Designing the Random ssDNA Library and Primers

The random ssDNA library and primers were purchased from Integrated DNA Technology (IDT, Coralville, IA, USA). The sequence of the random ssDNA library is 5′-TAATACGACTCACTATAGGAGCTCAGCCTTCACTGC-(N60) GGCACCACGGTCGGATCCAC-3′. N60 represents the length of the randomized region of the ssDNA library, while the underlined region is the T7 promoter sequence. ‘N’ can be either A, C, G, or T. The forward primer is 5′-TCTAATACGACTCACTATAGGAGCTCAGCCTTCACTGC-3′, while the reverse primer is 5′-GTGGATCCGACCGTGGTGCC-3′. For the production of RNA molecules for the first cycle of SELEX, the random ssDNA library was first PCR-amplified, followed by *in vitro* transcription.

### 2.2. PCR Amplification

PCR amplification was carried out in a 100 μL reaction mixture containing 0.2 mM dNTPs (Promega, Madison, WI, USA), 1X PCR buffer (Yeastern Biotech Co., Taipei, Taiwan), 2 µM forward reverse, 2 µM reverse primer and 5 U *YEAtaq* DNA Polymerase (Yeastern Biotech Co., Taipei, Taiwan). PCR amplification was executed using Biorad MyCyler Thermal Cycler and the parameters for PCR cycles were as follows: an initial denaturation at 95 °C for 60 s, amplification cycles that consist of 30 s denaturation at 95 °C, 30 s annealing at 55 °C, 30 s extension at 72 °C and final elongation of 120 s at 72 °C. The amplification was carried out starting with 6 PCR cycles and additional 2 cycles were added until the PCR band with the correct size appeared in each SELEX cycle. The resulting PCR product was verified by 4% agarose gel electrophoresis, with the size ascertained by using a 25 bp DNA ladder (Promega Corporation, Madison, WI, USA). Following the size verification, the PCR product was ethanol-precipitated.

### 2.3. In Vitro Transcription

Four microliters of the ethanol-precipitated PCR product were used as the template for the *in vitro* transcription reactions. *In vitro* transcription was carried out using Ampliscribe™ T7-Flash™ Transcription kit (Epicentre, Madison, WI, USA). The *in vitro* transcription reaction mixture was assembled in a 20 μL reaction mixture containing 1X AmpliScribe T7 reaction buffer (40 mM Tris-HCl [pH 7.5], 6 mM MgCl_2_, 10 mM NaCl, 2 mM spermidine), 7.5 mM each of ATP, CTP, UTP and GTP, 10 mM of dithiothreitol (DTT), 20 U of AmpliScribe^TM^ T7 Polymerase Flash Enzyme Solution (10 U/μL) and 20 U of RiboGuard RNase Inhibitor (40 U/μL). The mixture was then incubated at 37 °C for 16 h in a water bath (Fisher Scientific International, Inc., Hampton, VA, USA). Next, treatment with 10 U of RNase-Free DNase I (Fermentas, Burlington, ON, Canada) was conducted for 20 min at 37 °C. The *in vitro* transcription reaction was stopped by adding an equal volume of 2X RNA loading dye (8 M urea, 0.05% bromophenol blue), and the mixture was heated at 95 °C for 2 min in a thermomixer (Eppendorf, Hamburg, Germany). The *in vitro* transcription product was purified following electrophoresis on a 12%, 7 M denaturing urea polyacrylamide gel using the method described by Citartan et al., 2012 [10].

### 2.4. SELEX

A reaction mixture that comprised 6.6 µM of initial RNA pool in 1X SELEX binding buffer (150 mM NaCl and 10 mM HEPES-KOH [pH 7.4]) (GE Healthcare Bio Sciences, Uppsala, Sweden) was prepared. The mixture was heated for 2 min at 95 °C followed by cooling at RT for 10 min. Next, 10.2 µM yeast tRNA (Invitrogen Corporation, Carlsbad, CA, USA) was added as a competitor, followed by the addition of 2.5 µM of dengue virus serotype 2 NS1 protein (Abcam, Thailand). The reaction mixture was incubated at RT for 15 min. The concentrations of the RNA pool, yeast tRNA and the target NS1 protein used in every SELEX cycle were varied. Nitrocellulose filter membrane was used for partitioning for SELEX cycles 1, 2, 4, 6, 7, 9 and 10. On the other hand, partitioning for SELEX cycles 3, 5, 8 and 11 was aided by the Xenobind plate (ELISA microtiter plate) (Xenopore, Hawthorne, NJ, USA). The target-bound RNA molecules were recovered by heating at 95 °C with 7 M urea for 2 min before being ethanol-precipitated in the presence of Dr. GenTLE™ precipitation carrier. The eluted RNA molecules were subjected to reverse transcription using RevertAid First Strand cDNA Synthesis Kit (Thermo Fisher Scientific, Waltham, MA, USA), by following the manufacturer’s instructions. The resulting cDNA molecules from the reverse transcription reaction were PCR-amplified, ethanol-precipitated and used for *in vitro* transcription to prepare the RNA molecules for the next round of SELEX. After 11 cycles of SELEX, the binding of the RNA pool to the target NS1 protein was monitored by using a nitrocellulose filter-binding assay.

### 2.5. Nitrocellulose Filter Binding Assay

Prior to the labelling of the RNA molecules at the 5′-end with (γ-^32^P)-ATP, dephosphorylation was carried out using alkaline phosphatase enzyme (New England Biolabs, Massachusetts, USA) by following the manufacturer’s instructions. The reaction mixture was incubated at 37 °C for 60 min before ethanol precipitation using Dr.GenTLE^TM^ precipitation carrier. The dephosphorylated RNA was added to a reaction mixture containing 1X T4 RNA ligase reaction buffer A (Thermo Fisher Scientific, Waltham, MA, USA), 6.6 pmol of radiolabelled (γ-^32^P)-ATP (Perkin Elmer, Waltham, MA, USA) and 10 U of T4 polynucleotide kinase (Promega, Madison, WI, USA). The mixture was incubated at 37 °C for 60 min in a water bath. The radiolabelled RNA was purified using Quick spin Sephadex G-25 columns (Roche Diagnostics GmbH, Mannheim, Germany). The RNA pool/RNA aptamer candidates were heated at 95 °C for 2 min followed by cooling at RT for 10 min. To each of the RNA reaction mixtures, 200 nM of yeast tRNA and dengue NS1 protein were added to a final volume 50 µL, and they were incubated for 15 min at RT. The concentrations of the NS1 protein for all the 5th, 8th and 11th cycles of SELEX were 0, 800 and 1600 nM. On the other hand, for the binding evaluation of the RNA aptamer candidates, the concentrations of the NS1 protein were varied from 0 to 800 nM. For the total RNA, the reaction mixture, which contains only the radiolabelled RNA was added on top of the filter without any filtration. The total RNA serves as the denominator (100%), used to estimate the fraction of the total RNA bound at certain protein concentrations. All the other reaction mixtures were filtered through pre-wet nitrocellulose filter membranes under vacuum suction and were washed with 1 mL of 1X HEPES buffer. As a result, nitrocellulose membranes, which have the ability to retain protein by hydrophobic interaction, retained the protein together with the bound RNA molecules, while the unbound RNA molecules were washed away with the aid of vacuum suction. Following the filtration and washing, the filter membranes were collected and exposed to X-ray film before imaging using autoradiography [11,12,13]. Following autoradiography, the signal intensities from the nitrocellulose filter membranes were quantified using the ImageJ software (downloaded at https://imagej.nih.gov/ij/, accessed on 12 March 2021). Signal intensities obtained were used to estimate enrichment. Enrichment refers to the fraction of the total RNA bound at a specific concentration of the protein. It can be estimated by measuring the signal intensity that represents the fraction of the RNA bound to the protein at a specific concentration after the subtraction of the signal intensity at 0 nM protein concentration, which is the background reading. This obtained value was then divided by the total RNA intensity.

Enrichment = [ **(** (Bound RNA intensity – Background intensity)/ Total RNA intensity **)** ] × 100

The enrichment data obtained were then used to determine the equilibrium dissociation constant, *K*_d_ from a nonlinear regression curve using the GraphPad Prism software (version 6.05) (GraphPad Software Inc., San Diego, CA, USA).

### 2.6. Cloning, Transformation and Sequence Analysis

The RNA pool after the completion of the SELEX cycles was subjected to PCR amplification, with the extension time extended up to 10 min. The resulting PCR product was cloned into pCR@2.1 cloning vector using TOPO TA cloning kit (Thermo Fisher Scientific, Waltham, MA, USA). After transformation was carried out using the chemically competent *Escherichia coli* (TOP 10 strain) cells, blue–white screening was performed and a total of 28 white colonies (positive clones) were randomly picked and subjected to plasmid extraction using High Pure Plasmid Isolation Kit (Roche Diagnostics GmbH, Mannheim, Germany). After confirming the quality of the extracted plasmids, sequencing was carried out in First BASE Laboratories Sdn. Bhd., Selangor, Malaysia. RNA sequences were derived from the DNA sequences. The sequences obtained were analysed for sequence homology using CLUSTALW (http://www.ebi.ac.uk/clustalw/, accessed on 26 March 2021). Identical sequences that appeared more than once were clustered together to represent a class or cluster of that particular sequence. The percentage of appearance of a particular sequence is the total number of times a particular sequence appears per the total number of all the sequences, expressed in the form of a percentage. All the sequence clusters were subjected to binding evaluation to the target NS1 protein.

### 2.7. Secondary Structure Determination and the Truncation of the Aptamers

The secondary (2D) structures of the full-length DENV aptamers were predicted using RNAfold of the Mfold programme with default settings using the Zuker algorithm [14]. The secondary structure with the lowest Gibbs free energy (dG) was chosen as the most accurate conformation for each full-length RNA aptamer. Following the prediction, each of the full-length aptamer sequences was subjected to a rational truncation approach, whereby nucleotides were removed to produce the shortest aptamer variant that can still retain their core secondary structures. These truncated versions of each aptamer were further subjected to *in silico* analysis.

### 2.8. Conversion of the RNA Sequences from 2D to 3D Structures

The tertiary (3D) structures of the truncated RNA aptamers were modelled by first reidentifying and confirming their 2D structures using the RNAfold of Mfold. The secondary structure with the lowest Gibbs free energy (dG) was chosen as the most accurate/preferred conformation. Each folded sequence was then converted to the Vienna format, which is a representation of secondary structures in the form of dots and brackets. The resulting secondary structures were then fed into RNAComposer (rnacomposer.cs.put.poznan.pl, accessed on 7 March 2022) using the default settings to convert the 2D structures to 3D PBD coordinate files [15].

### 2.9. Rigid Molecular Docking

For rigid molecular docking, three different docking software were utilized. The aptamers were treated as the ligands for each docking submission, while the target dengue NS1 was assigned as the receptor molecule. Docking using AutoDock Vina (downloaded using the link vina.scripps.edu) was conducted under high-performance computing (HPC) environments with default parameters and a grid box with the size of 126, 126, 126 encompassing the entire NS1, centred at −8.7, −29.33 and −27.58, corresponding to the x, y and z dimensions, respectively. HADDOCK (available at alcazar.science.uu.nl/services/HADDOCK2.2, accessed on 13 March 2022) was also performed with default settings, using the active and passive residues of the NS1 protein predicted by Cport (available at alcazar.science.uu.nl/services/CPORT, accessed on 15 March 2022). PatchDock webserver (available at bioinfo3d.cs.tau.ac.il/PatchDock, accessed on 18 March 2022) was applied using the aptamers as the ligand and Dengue NS1 proteins as the receptor files with the default values of 4.0 for clustering RMSD and default complex type. For PatchDock, the ten best PatchDock results of the docked structures were further refined using FireDock options provided on the docking results page.

### 2.10. Molecular Dynamic (MD) Simulation of the RNA Aptamer–NS1 Protein Complex

MD simulations were conducted using the GROMACS program (downloaded at gromacs.org, accessed on 2 April 2022), in which Amber99SB-ILDN force field parameters for RNA–protein complex simulations were applied. The complexes were solvated using TIP-3 water models in a cubic box, maintaining a centric position and keeping a distance of 1.0 nm between the complex and the edge of the solvated box. Sodium and chloride ions were added to neutralize the charge of the entire system. The Particle Mesh Ewald (PME) method was applied to simulate electrostatic interactions. Energy minimization steps were followed through using the steepest descent algorithm with the tolerance of 1000 kJ mol^−1^nm^−1^. The Verlet cutoff scheme was chosen, and periodic boundary conditions were assigned to all three dimensions (XYZ). Both thermal (NVT) and subsequent pressure equilibration (NPT) phases were simulated for 0.1 ns with a 2 fs integration/time step. NVT was conducted at room temperature (300 K), while NPT was performed using the Berendsen pressure coupling scheme. Both equilibrations were carried out using velocity-rescale temperature coupling by assigning the RNA–protein and water ions as individual groups. All bond lengths were constrained by employing LINCS algorithms. Final MD simulations of each complex were performed for 50 ns at equilibrated temperature and pressure. The best possible structure or trajectory frame for each complex of MD simulation was chosen using cluster command analysis of the coordinate file by selecting the structures with the largest cluster size and lowest RMSD values among the time frames (ns). GROMACS command tools were used to analyse the Rg values of the chosen RNA–protein complex structure.

### 2.11. Functionalization of the RNA Aptamer with Biotin at the 3′-End for ELASA

The full-length aptamers were functionalized with poly(A) tail at the 3′-end by PCR amplification using the forward primer, 5′-TCTAATACGACTCACTATAGGAGCTCAGCCTTCACTGC-3′ and the reverse primer, 5′-TTTTTTTTTTTTTTTTTTTTTTTTGTGGATCCGACCGTGGTGCC-3′, followed by *in vitro* transcription and purification. For the preparation of the truncated RNA aptamers, the forward and reverse PCR primers were designed manually for the PCR amplification of the DNA templates used for the subsequent *in vitro* transcription reactions (sequences enlisted in Supplementary Materials Table S1). The forward primers for each different truncated aptamer were incorporated with a T7 RNA polymerase promoter sequence at the 5′ end (5′-TCTAATACGACTCACTATA-3′), while the reverse primers were incorporated with a poly(dT) tail extension at the 5′ end (5′-TTTTTTTTTTTTTTTTTTTTTTTT-3′).

### 2.12. In Vitro Transcription of the Stabilized Truncated RNA Aptamers

Four microliters of the ethanol-precipitated PCR product were *in vitro*-transcribed using DuraScribe T7 Transcription Kit (Lucigen, Middleton, WI, USA) to prepare the stabilized truncated RNA aptamers. The reaction mixture was assembled using 1X DuraScribe T7 Reaction Buffer (40 mM Tris-HCl [pH 7.5], 6 mM MgCl_2_, 10 mM NaCl, 2 mM spermidine), 7.5 mM of each ATP, GTP, 2′-F-dCTP and 2′-F-dUTP, 10 mM of dithiothreitol (DTT), 20 U of DuraScribe T7 Enzyme Mix (10 U/μL) and 20 U of RiboGuard RNase Inhibitor (40 U/μL), to a final volume of 20 μL. The mixture was then incubated at 37 °C for 16 h. The *in vitro* transcription product was purified following electrophoresis on a 12%, 7 M denaturing urea polyacrylamide gel.

### 2.13. Direct ELASA

The target NS1 protein of various concentrations (3–100 nM) was dissolved in a PBS buffer (pH 7.4) and coated onto ELISA microtiter plate wells, incubated overnight at 4 °C. The unbound proteins were removed by washing the wells three times using 1X PBST, followed by blocking with 3% BSA in Superblock buffer for 2 h at 37 °C. After washing thrice, one hundred picomoles of the RNA aptamer appended with poly(A) tail at the 3′-end was mixed with the biotinylated poly(dT)_20_ in 1X LINA buffer (150 mM LiCl and 150 mM NaCl in dH_2_O) and were heated at 95 °C for 2 min followed by cooling to room temperature (RT) for 15 min to promote folding and duplex formation. The biotin-functionalized aptamers were then added into the wells and incubated for 1 h at RT. Washing with 1X LINA-T (LINA with 0.05% Tween-20) was carried out thrice to remove the unbound aptamers before the addition of 100 µL of streptavidin–HRP diluted with a dilution factor of 1:1000. After washing thrice to remove the unbound streptavidin–HRP, 100 microliters of TMB substrate was subsequently added and incubated in the dark for 20 min, followed by the addition of 100 µL of 1 M HCL solutions to stop the reactions. ELASA reactions were conducted in triplicate. All OD measurements were taken using an ELISA plate reader at 450 nm.

#### 2.13.1. Cross-Reactivity Analyses

The cross-reactivity of the selected aptamer candidates to other proteins was verified using direct ELASA with the recombinant Envelope 2 (E2) protein of the Chikungunya virus, the LipL32 protein of *Leptospira* bacteria and the NS1 protein of Zika. Equal concentrations of 100 nM of the E2 and Lipl32 proteins were dissolved in a PBS buffer (pH 7.4) and coated onto individual microtiter-plate wells, left for incubation overnight at 4 °C. Following the coating of the proteins onto the wells, the following steps were carried out as described previously.

#### 2.13.2. Limit of Detection (LOD)

The minimum amount of the target NS1 dengue that can be detected by the system, the limit of detection or LOD was determined. The NS1 protein was dissolved in a coating buffer using different dilutions of commercial human serum. Three different dilutions of the commercial human serum (Sigma-Aldrich, Rockland, MA, USA) were prepared using 1X PBS (pH 7.4) buffers. These dilutions were pure serum (100%), 1:100 (1%) and 1:1000 (0.1%). NS1 at a concentration of 100 nM was dissolved in a PBS buffer (pH 7.4) and coated onto three individual ELISA microtiter plate wells, left for incubation overnight at 4 °C. The wells were washed three times with 1X PBST to remove the unbound proteins. Each of the diluted serum solutions was incubated in the individual microtiter plate wells (pre-coated with NS1) and left for incubation overnight at 4 °C. The wells were again washed three times with 1X PBST to remove the unbound proteins. Subsequently, the following steps were carried out as described previously.

#### 2.13.3. Direct ELASA of Stabilized TDENVs at Different Times of Incubation

One hundred nanomolar (100 nM) of the NS1 protein was prepared in a PBS buffer (pH 7.4) and coated onto the ELISA microtiter plate wells, incubated overnight at 4 °C. The unbound proteins were removed by washing the wells three times using 1X PBST, followed by blocking with 3% BSA in Superblock buffers for 2 h at 37 °C. After washing thrice, one hundred picomoles of the unstabilized and stabilized TDENV aptamers appended with poly(A) tail at the 3′-end, each mixed with biotinylated poly(dT)_20_ in 1X LINA buffer, were heated at 95 °C for 2 min followed by cooling to room temperature (RT) for 15 min. The biotin-functionalized aptamers were then added into the wells and incubated for 15, 30 and 60 min. Following aptamer incubation and washing, the following steps were carried out as described previously.

### 2.14. Sandwich ELASA

Sandwich ELASA was performed by coating the ELISA microtiter plate wells with 300 picomoles of the folded TDENV-3 RNA (capture probes) prepared in 1X PBS buffer and allowed to incubate for one hour. The unbound aptamers were removed by washing three times using 1X PBST followed by incubation with different concentrations of the target NS1 protein dissolved in PBS buffer. After 1 h, the wells were again washed three times with 1X PBST. One hundred picomoles of the TDENV-6a RNA aptamers appended with poly(A) tail at the 3′-end and biotinylated poly(dT)_20_ dissolved in 1X LINA buffer were heated at 95 °C for 2 min followed by cooling to RT for 15 min. The biotin-functionalized TDENV-6a aptamers (reporter probes) were then added into the wells and incubated for 1 h at RT. The wells were washed three times using 1X LINA-T before the addition of 100 µL of streptavidin–HRP prepared using the dilution factor of 1:1000. After washing, 100 microliters of TMB substrate were subsequently added and incubated in the dark for 20 min, followed by the addition of 100 µL of 1 M HCL solutions to stop the reactions. OD measurements was carried out using an ELISA plate reader at 450 nm.

#### 2.14.1. Sandwich ELASA with Cumulative Incubations

Sandwich ELASA was performed by coating the ELISA microtiter plate wells with 300 picomoles of the folded stabilized TDENV-3 RNA (capture probes) dissolved in 1X PBS buffer and allowed to incubate for one hour. The unbound aptamers were removed by washing three times using 1X PBST. The NS1 protein of different concentrations were incubated repeatedly 8 times, with each incubation lasting for 15 min. Following repeated incubation of the proteins, the following steps were carried out exactly as described previously.

#### 2.14.2. Sandwich ELASA in Serum Diluents

Different dilutions of commercial human serum were prepared using 1X PBS (pH 7.4) buffers using the dilution factors of 1:100, 1:1000, 1:2000, 1:5000 and 1:10,000. Sandwich ELASA was performed by coating the ELISA microtiter wells with 300 picomoles of the folded stabilized TDENV-3 RNA in 1X PBS buffer and allowed to incubate for one hour. The unbound aptamers were removed by washing three times using 1X PBST followed by incubation with the target NS1 protein of different concentrations spiked into the serum prepared with different dilutions. The incubation was carried out for 1 h. The wells were again washed thrice with 1X PBST. One hundred picomoles of both the stabilized TDENV-6a RNA aptamers appended with poly(A) tail at the 3′-end and biotinylated poly(dT)_20_ were dissolved in 1X LINA buffer. The mixture was heated at 95 °C for 2 min followed by cooling to RT for 15 min. The biotin-functionalized, stabilized TDENV-6a aptamers (reporter probes) were then added into the wells and incubated for 1 h at RT. Following the addition of the reporter probes, the following steps were carried out as described previously.

### 2.15. Statistical Analysis

All experiments were performed in replicate to analyse the mean and standard deviations of the obtained raw data. Independent *t*-tests were conducted using GraphPad prism. Alpha value was set at 0.05 and the statistical significance was determined as *p* < 0.05.

## 3. Results and Discussion

### 3.1. Nitrocellulose Filter Binding Assay Suggests Enrichment of the Nucleic Acid Pool from the 11th Cycle of SELEX

**Figure 1:**
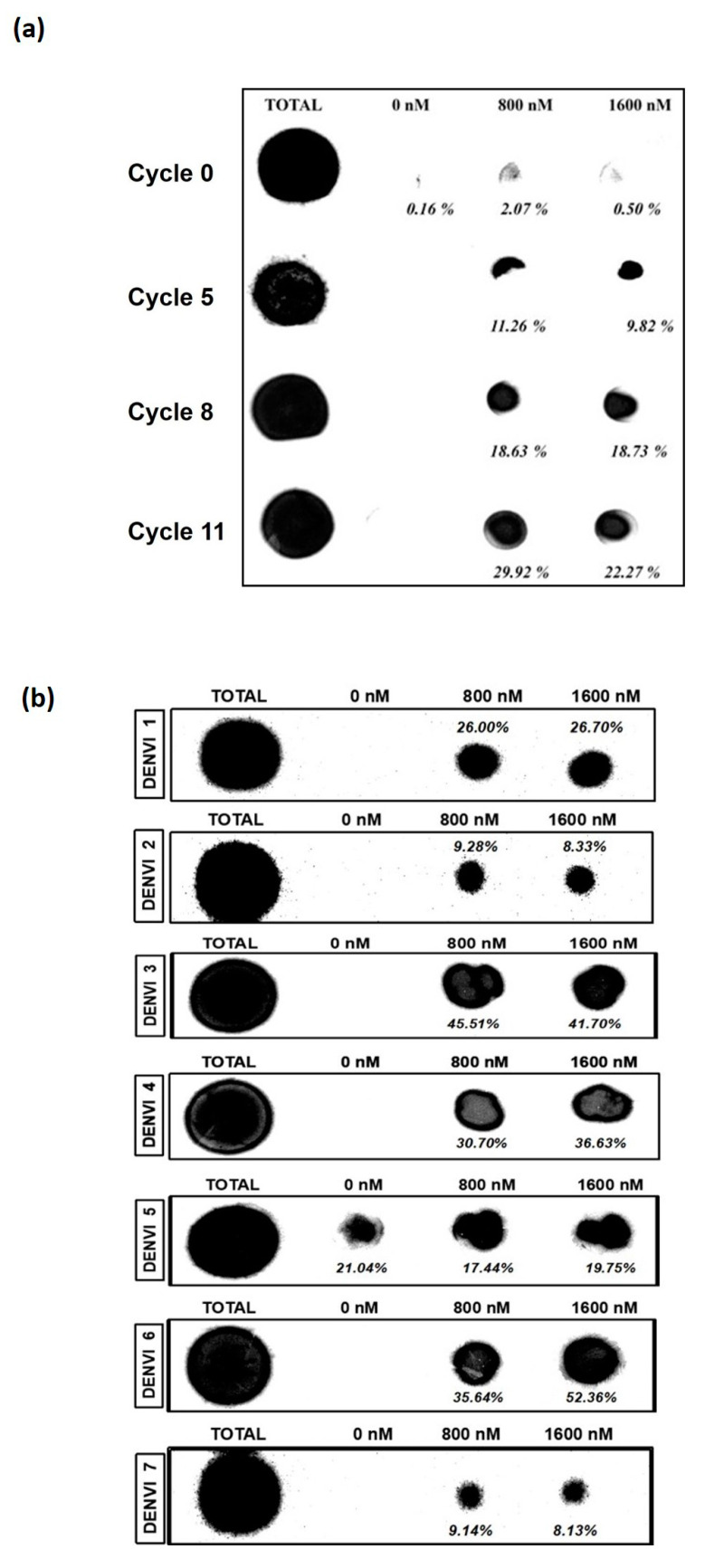
(**a**) Nitrocellulose filter binding assay of the RNA pool to NS1 protein. The RNA pool used was the initial randomized RNA library, RNA pool from the 5th cycle of SELEX, RNA pool from the 8th cycle of SELEX and RNA pool from the 11th cycle of SELEX. (**b**) Nitrocellulose filter binding assay of seven sequence clusters to the target protein NS1. ‘Total’ represents the total RNA. Dot visualized at the corresponding concentrations of NS1 represents the fraction of the total RNA bound to the target NS1. The figures shown are the representative figures of independent experiments performed in duplicate.

In this study, a total of 11 cycles of SELEX were carried out. Throughout the SELEX process, selection pressure was exerted by reducing the amount of target protein and RNA pool with the concomitant increase in the amount of competitor yeast tRNA (Table 1). In this study, the binding of the nucleic acid pool from SELEX cycles 0, 5, 8 and 11 were evaluated against the target dengue NS1. The enrichment of 11.26% and 9.82% was obtained at 800 and 1600 nM of the dengue NS1 protein by the nucleic acid pool from SELEX cycle 5, respectively. The enrichment increased to 18.63% and 18.73%, demonstrated by the nucleic acid pool from SELEX cycle 8, while the enrichment of 29.92% and 22.27% were estimated for the nucleic acid pool from cycle 11 at 800 and 1600 nM of the NS1 protein, respectively (Figure 1a). As the nucleic acid pool from SELEX cycle 11 achieved the highest enrichment, sequencing was carried out to identify the potential binders. The sequence analysis revealed seven clusters of sequences (named DENV-1 to DENV-7). The percentage of appearance for each sequence was calculated (Table 2). DENV-1, -3, -4 and -5 had the highest percentage of appearance, with a percentage of 17.86%. DENV-2 and DENV-6 had a percentage of appearance of 10.71%, while DENV-7 had the value of 7.14% (Table 2).

### 3.2. DENV-3 and -6 Are the Most Potent Aptamers

**Figure 2:**
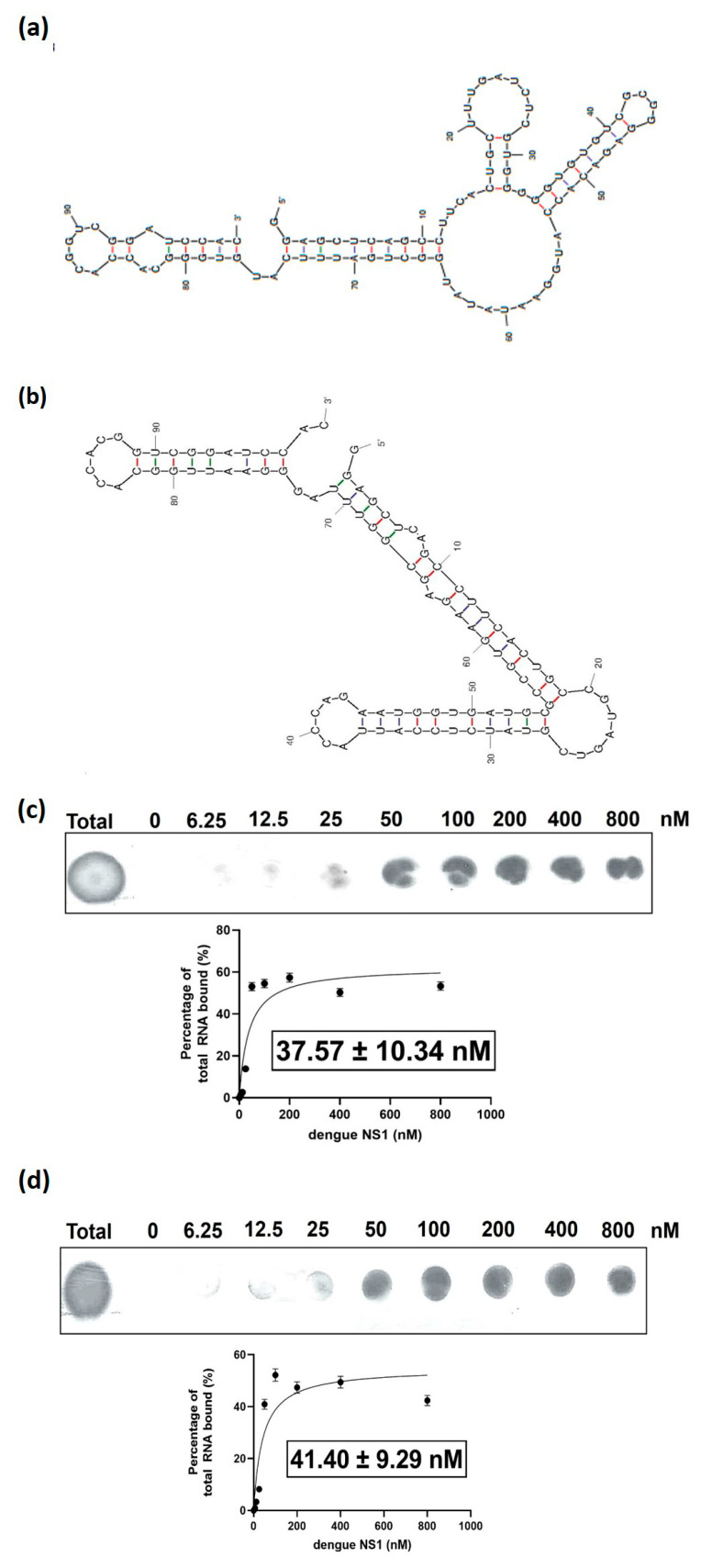
(**a**) The secondary structure of DENV-3 aptamer (**b**) The secondary structure of DENV-6 aptamer (**c**) Determination of equilibrium dissociation constant, *K*_d_, of the DENV-3 aptamer to the NS1 by titration of a fixed concentration of ^32^P-labelled RNA aptamer against different concentrations of NS1 (0–800 nM). The value of 37.57 ± 10.34 nM represents the concentration of protein at which 50% of the radiolabelled DENV-3 aptamer is in the form of DENV-3-NS1 complex. (**d**) Determination of equilibrium dissociation constant, *K*_d_, of the DENV-6 aptamer to the NS1 by titration of a fixed concentration of ^32^P-labelled RNA aptamer against different concentrations of NS1 (0–800 nM). The value of 41.40 ± 9.29 nM represents the concentration of protein at which 50% of the radiolabelled DENV-6 aptamer is in the form of DENV-6–NS1 complex. The figures shown are the representative figures of independent experiments performed in duplicate.

All the seven classes of sequences obtained following the sequencing of the nucleic pool from the 11th SELEX cycle were subjected to a nitrocellulose filter-binding assay. DENV-3 and DENV-6 were chosen as the most potent aptamers as they show the highest enrichment among all the classes of sequences, at 800 and 1600 nM (Figure 1b). DENV-2 and DENV-7 were ruled out, as those candidates possess the lowest enrichment at 800 and 1600 nM. Although DENV-5 also showed considerable binding, the binding manifesting at 0 nM implies that this particular sequence exhibits nonspecific binding or acts as a filter binder that binds to the surface of the nitrocellulose filter membrane via weak electrostatic attraction. The secondary structures of DENV-3 and -6 are shown in Figure 2a,b. DENV-3 and DENV-6 were selected for equilibrium dissociation constant value, K_d_, determination.

### 3.3. Estimation of the Equilibrium Dissociation Constant, K_d_, of DENV-3 and DENV-6 RNA aptamers

The equilibrium dissociation constant of the aptamer DENV-3 and DENV-6 was estimated via a nitrocellulose filter membrane assay. By using nonlinear regression analysis via GraphPad Prism, the values were estimated at 37.57 ± 10.34 nM and 41.40 ± 9.29 nM for DENV-3 and DENV-6, respectively (Figure 2c,d). These aptamers exhibited different affinities due to the presence of different epitopes on the surface of the target protein that could result in various strengths of binding contributed by hydrophobic, van der Waals, hydrogen bond and electrostatic interactions. The potential of these epitopes to establish interactions is also corroborated by the binding nature of the NS1 protein during dengue virus infection. During the viral infection, the NS1 protein interacts with the NS4B viral protein and ER lumen in the host cell, which lead to the viral replication of the dengue virus [16]. Thus, the natural affinity of the NS1 protein to multiple interacting partners also permitted the isolation of different aptamers with various *K*_d_ values during the SELEX process.

### 3.4. Rational Truncation of the Full-Length DENV Aptamers to Truncated Forms 

**Figure 3:**
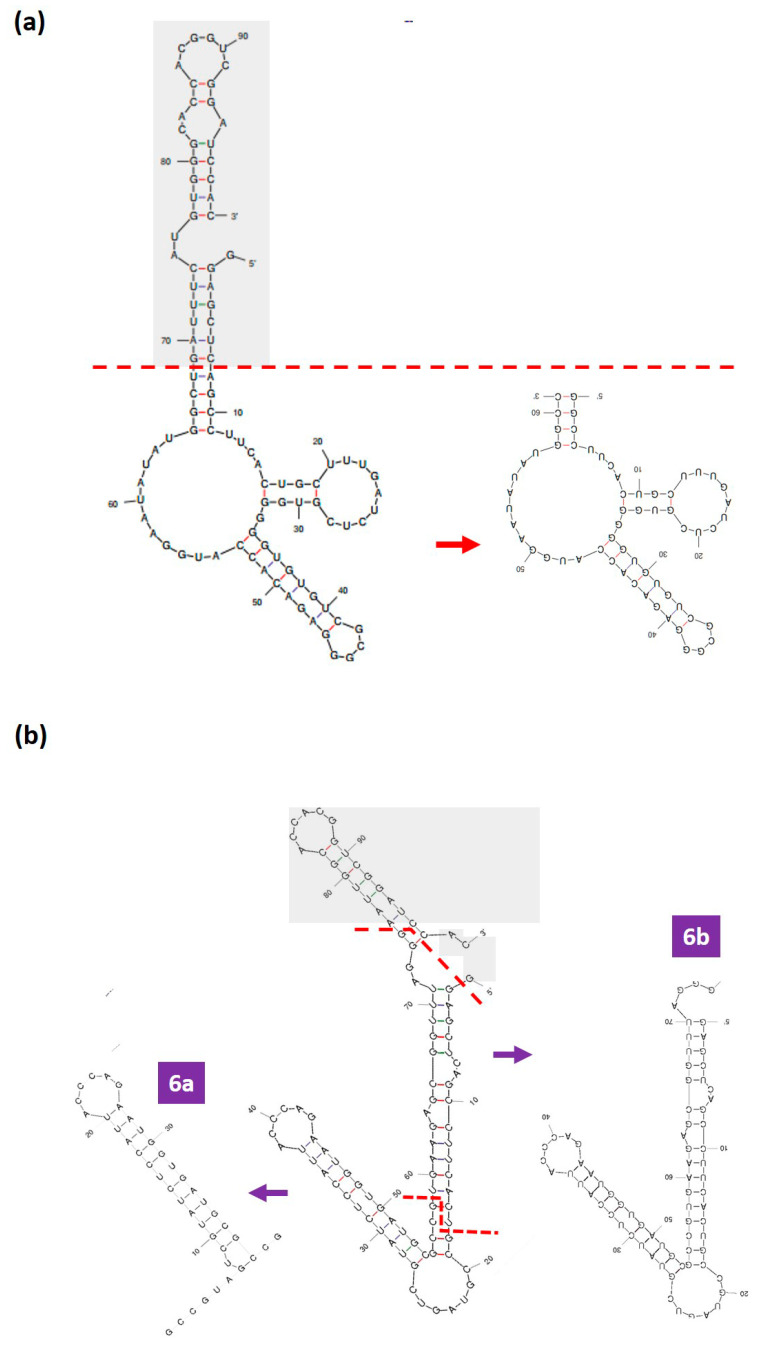
(**a**) Rational truncation of full-length DENV-3 aptamer to TDENV-3 aptamer. The grey box highlights the region of the aptamer which was removed during truncations. The red dotted line separates the region of the aptamer chosen for rational truncation. (**b**) Rational truncation of full-length DENV-6 aptamer to TDENV-6a and TDENV-6b aptamer. The red dotted line separates the region of the DENV-6 aptamer chosen for truncation.

In this study, we hypothesized that the truncation/shortening of the full-length DENV aptamers into shorter versions that still retain the potential core secondary structures may improve their binding affinities. The elimination of the excessive sequence regions that potentially contribute to electrostatic repulsions while retaining the core secondary structures can improve the binding affinity of the truncated aptamers compared to their full-length versions. The core secondary structures refer to the structural motifs that are highly likely to harbour affinities against the target protein, such as the well-defined loop region. Secondary structures such as the loop region are the binding hotspot of the aptamers to their cognate target [17,18], while the stem region can act as an element that can support the loop region. The procedure of aptamer truncation adopted in this study is the rational truncation approach that involves the removal of excess nucleotides such as the stem region, which is double-stranded and is less likely to be involved in the binding to the target aptamer. However, the removal of the stem structure must be carried out with caution as part of the stem region may also be useful to support the loop region of the aptamers [19,20]. After the removal, the truncated aptamers can always be refolded using the Mfold programme to check the retention of the core secondary structures. Any disruption in the core secondary structure indicates the possible debilitation of the binding of the aptamer to its target NS1. The full-length DENV-3 aptamer was truncated by retaining a rationally selected nucleotide region/portion (8–68) while removing the remaining parts, resulting in TDENV-3 (Figure 3a). The A nucleotide at the position 8 was replaced with a G residue while the U residue at the position 68 was replaced with G, so that a G-C pairing can be formed and this potentially stabilizes the miniaturized structure. This shortened sequence was again re-examined using the Mfold programme and it can be observed that the core secondary structures present in the full-length version were retained. Similarly, for DENV-6, nucleotide regions (18–58) and (2–75) were chosen to derive two different truncated versions, designated TDENV-6a and TDENV-6b, respectively. These two variants are also able to retain their core secondary structures present in their full-length versions (Figure 3b). TDENV-6a and TDENV-6b were subjected to *in silico* binding analysis.

### 3.5. Truncated Aptamers Display Higher Binding Scores Compared to Full-Length Aptamers, Suggesting an Improvement in their Binding Strength

For rigid docking, three different docking softwares were used; Autodock Vina, HADDOCK and Patchdock. Autodock Vina performs molecular docking by providing several binding modes ranked based on their global free energy. HADDOCK applies ambiguous interaction restraints (AIR) by establishing atomic distance restraints between predefined active and passive residues under multiple iterations. HADDOCK scores are then assigned to distinguish between the correct and flawed docking models. Patchdock generates binding assemblies by relying on molecular shape complementarity using Connolly’s solvent accessible surface areas [21,22,23,24]. Utilizing more than one docking function enables us to cumulatively capitalize the advantage of each scoring matrix, resulting in scores that are more accurate, representative and reliable than that which can be achieved using a single docking software.

Each docking software predicts several possible RNA–protein complex structures. The docking scores of the top 10 binding models of all the aptamer–NS1 complexes (of both the truncated and the full-length aptamers) predicted by each docking software were recorded. The top 10 binding scores were statistically normalized by computing the Z-score value using Equation (1), where E is the highest binding score, Ē is the mean binding score and σ is the standard deviation. A total Z-score (Z_T_) value was computed from the sum of the three Z-score derivatives of each docking method obtained for each of the aptamer sequences, as shown in Table 3.


(1) $$Z\;=\;(E-\overset{–}{E})\;/\;\sigma$$

From the docking results, it was apparent that the truncated aptamers were predicted to have stronger binding to their target NS1 compared to the full-length aptamers. This can be seen from Table 3, where the Z_T -_scores of TDENV-3 of −5.34 and TDENV-6a of −5.26 are higher compared to the Z_T_ scores of their full-length versions, which are −4.24 and −4.83, respectively. However, the docking score of 6b is −4.77, which is lower compared to its full-length version, which has a score of −4.83. The validity of the docking approach was also evaluated in parallel using control sequences for docking against the target NS1 (Table 4). We observed a statistically significant difference between the truncated aptamers (TDENV-3 and -6a) and the control sequences (*p* = 0.006, *α* = 0.05). This corroborates the reliability of the obtained Z_T_ values as an efficient scoring metric in evaluating the binding of the aptamers to their target NS1.

### 3.6. Lower Rg Values Corroborate Better Binding of the Truncated Aptamers Compared to Full-Length Aptamers

**Figure 4:**
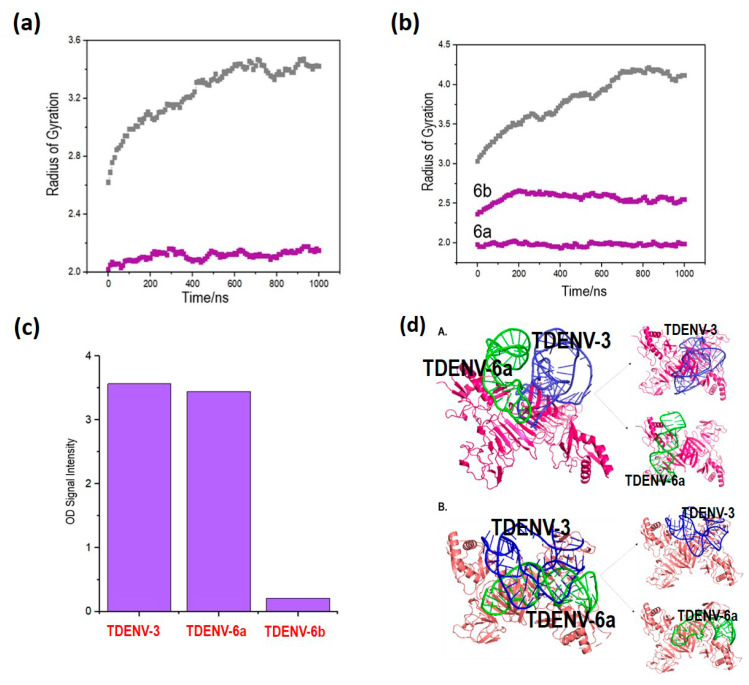
(**a**) The radius of gyration, Rg values for TDENV-3 (purple-coloured line) and full-length DENV-3 (grey-coloured line) aptamers. (**b**) The radius of gyration, Rg values for TDENV-6a and TDENV-6b (purple-coloured line) and full-length DENV-6 (grey-coloured line) aptamers. (**c**) OD readings of the binding of the truncated aptamers to the dengue NS1. (**d**) TDENV-6a docked to pre-docked TDENV-3/NS1 complex. Dual-docked model variants from (A) Patchdock and (B) Haddock software.

The docked models of the aptamer–NS1 complex (both truncated and full-length) with the highest Z-score were subsequently analysed by measuring their Rg values using a 1 ns MD simulation. The truncated aptamers exhibit lower Rg values compared to their full-length versions, implying stronger binding (Figure 4a,b). A lower Rg value implies the ability of the aptamers to forge tighter interaction with their target due to the binding-induced conformational changes [25]. It can also be conjectured that the truncated aptamers assume alternative or more distinctive conformations, which are much more compact or tighter upon binding to NS1, strongly suggesting target-induced conformational changes that are more pronounced than that demonstrated by the full-length aptamers [26].

### 3.7. TDENV-3 and -6a as Potential Aptamers against Dengue NS1 as Corroborated by Direct ELASA

The binding of the truncated aptamers to dengue NS1 was analysed using NS1 target concentration of 100 nM. The results show that only TDENV-3 and -6a retained binding to NS1, while TDENV-6b shows no binding (Figure 4c). This is consistent with the results from the *in silico* analysis, which demonstrates that TDENV-6b shows poorer binding compared to its full-length version. Therefore, only TDENV-3 and -6a were chosen for further validation.

### 3.8. TDENV-3 and TDENV-6a Aptamers Occupy Distinct Epitopes of NS1, Suggesting Their Usage in Sandwich Assays

TDENV-3 and TDENV-6a aptamers were also docked concurrently with the NS1 target to identify the possibility for the hetero-cooperative binding of the aptamers at distinct sites. The previously docked models of the TDENV-3–NS1 complex acquired from Haddock and Patchdock were further docked with TDENV-6a aptamers. Following dual docking, we can observe that both the TDENV-3 and TDENV-6a display binding to distinct sites of NS1. Based on the docking results, the dual interaction of both TDENV-3 and -6a structures to NS1 are visibly distinguishable, as seen in Figure 4d. The Z-score values of TDENV-6a docked with pre-complexed TDENV-3–NS1 are also significant compared to the previously computed Z-score values of TDENV-6a and TDENV-3 independently docked with NS1 (Table 5). This ability of the truncated aptamers to bind to distinct epitopes of the NS1 target can potentiate the development of sandwich assays.

### 3.9. Truncated Aptamers Have a Better LOD Compared to Full-Length DENV

**Figure 5:**
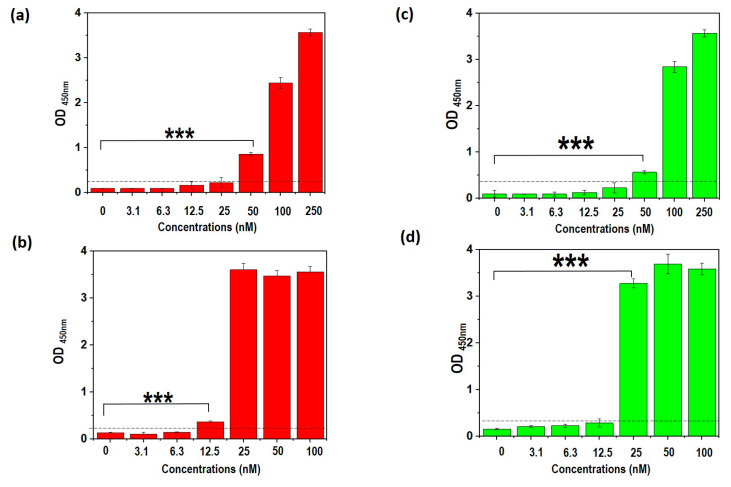
(**a**) Determination of the LOD of the truncated and full-length aptamers to the target NS1 (**a**) full-length DENV-3 and (**b**) TDENV-3 (**c**) full-length DENV-6 and (**d**) TDENV-6a (*** = *p* < 0.001). The figures shown are the representative figures of independent experiments performed in triplicate.

The limit of detection (LOD) of the selected TDENV-3 and -6a aptamers were determined relative to the full-length DENV aptamers using the dengue NS1 in the range of 3–100 nM. The LOD represents the minimum concentration of the target dengue NS1 protein that is statistically significant (α = 0.05) and higher than the OD reading of the background, which acts as the control (0 nM of NS1). As shown in Figure 5a, the full-length version of DENV-3 has a LOD of 50 nM while TDENV-3 was able to detect down to 12.5 nM NS1 (Figure 5b). Similarly, the full-length version of DENV-6a displays a LOD of 50 nM (Figure 5c), while TDENV-6a has a LOD of 25 nM (Figure 5d). Hence, it can be inferred that both the truncated aptamers are able to exhibit a much lower LOD as compared to the full-length aptamers. TDENV-3 is also a stronger NS1 binder compared to TDENV-6a, based on the much lower LOD observed for TDENV-3. The data correspond well with the *in silico* analysis, further corroborating the legitimacy of the *in silico* analysis performed to evaluate the binding of the aptamers to the target NS1.

### 3.10. TDENV-3 and -6a Show High Specificity to Dengue NS1

**Figure 6:**
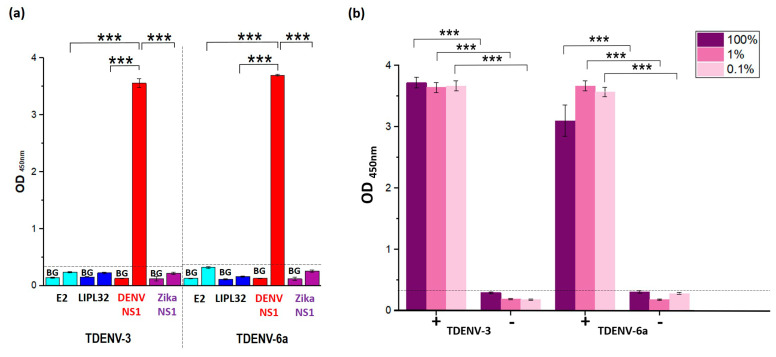
(**a**) Cross-reactivity analysis of TDENV-3 and TDENV-6a against NS1, E2 and LipL32 proteins together with their respective background readings (BG) (*** = *p* < 0.001). (**b**) OD readings that represent the binding of TDENV-3 and -6a aptamers to NS1 in different dilutions of serum. The ‘+’ indicates the presence of NS1 while ‘-’ indicates the absence of NS1 (*** = *p* < 0.001). BG refers to the background reading (protein concentration of 0 nM). The figures shown are representative figures of independent experiments performed in triplicate.

The two TDENV aptamers were further examined for their cross-reactivity to several other antigenic candidates. The target antigens used were the Lipl32 protein of *Leptospira*, the Envelope 2 (E2) protein of the Chikungunya virus and the NS1 protein of the Zika virus. The LipL32 and E2 are the membrane proteins of the causative agents of leptospirosis and Chikungunya viral infection, respectively, with clinical symptoms similar to dengue fever [27,28]. The NS1 protein of the Zika virus shares more than 75% sequence homology with the dengue NS1, as both belong to the same genus of Flavivirus. Hence, the NS1 protein of the Zika virus was chosen to investigate the cross-reactivity of the truncated aptamers. Figure 6a shows that the truncated aptamers produce a higher OD reading when tested against the dengue NS1, while no significant signal was produced with the other target proteins. The truncated TDENV-3 and -6a aptamers show highly specific binding to the target dengue NS1.

### 3.11. TDENV-3 and -6a Selectively Target NS1 in Human Serum

The diagnostic potentiality of aptamers is dependent on their capability to selectively bind to their target even in complex biological mixtures. In this study, human serum was used as the diluent in coating the wells of microtiter plates to simulate the complex *in vivo* cellular conditions and further verify the diagnostic potential of the TDENV aptamers. Here, we have utilized a stepwise manner of incubation to enable the better adsorption of the target NS1 to the surface of the wells. This was conducted by prior overnight incubation of wells with 100 nM of NS1, followed by secondary overnight incubation with the serum diluted using three different dilution factors (100% (pure), 1% and 0.1%). Empty wells were also coated with serum prepared using different dilution factors to measure the background reading (without NS1). The two-step strategy of first coating the target NS1 onto the wells of the microtiter plate enables more efficient adsorption of the target protein onto the wells, followed by the adsorption of the serum components. This allows the various molecules present in the serum to be also coated onto the surface of the wells alongside the target protein NS1. The spiking of NS1 into serum before coating can potentially result in a large number of interfering serum components that may compete with the target NS1 by clogging/reducing the surface area of the well accessible for the adsorption of the NS1. As a result, the coated NS1 can be much lower than the actual or the input concentration. As seen in Figure 6b, the OD values measured for both TDENV-3 and -6a in all three wells coated with NS1 serum (100%, 1% and 0.1%) were significantly higher compared to wells coated with only serum. The OD readings of the serum only prepared at different dilution factors (100%, 1% and 0.1%) for TDENV-3 and -6a equal the OD readings that represent the background signal measured during direct ELASA. This strongly suggests the ability of both the truncated aptamers to harbour high target selectivity to NS1, while exhibiting minimal to negligible levels of nonspecific binding to the serum components. These aptamers can be potentially used in detecting the target dengue NS1 in serum samples of infected patients with reduced false-positive results.

### 3.12. The Development of an Aptamer-Based Sandwich ELASA

**Figure 7:**
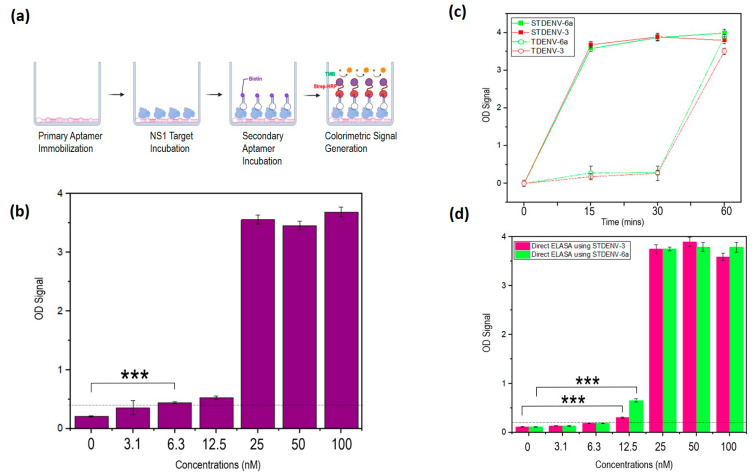
(**a**) Schematic representation of the developed sandwich ELASA platform (**b**) Determination of the LOD of the sandwich ELASA developed using TDENV-3 as the capture probe and TDENV-6a as the reporter probe (*** = *p* < 0.001). (**c**) OD readings of the binding of TDENV and STDENV aptamers at different time points. (**d**) Determination of the LOD of the direct ELASA using STDENV-3 (pink) and STDENV-6a (green) (*** = *p* < 0.001). The figures shown are representative figures of independent experiments performed in triplicate.

As TDENV-3 manifested a much lower LOD of 12.5 nM than TDENV-6a, it was used as the primary recognition element/capture probe for the target NS1, while the latter was used as the detection probe in the sandwich ELASA. This maximizes the capturing of the target NS1. The immobilization of the TDENV-3 capture probe to the surface of the well was achieved through passive adsorption. Although the aptamers were immobilized in random orientations, our findings suggest that the aptamers can still establish binding to NS1. This provides a cost-effective yet efficient method of immobilization using an unmodified aptamer rather than relying on streptavidin–biotin interaction. The TDENV-6a aptamer was synthesized to include a poly(A) tail at the 3′ end that enables the aptamers to form a duplex with the biotinylated complementary poly(dT). This allows the incorporation of biotin molecules into the aptamers, allowing the molecules to subsequently bind to streptavidin–HRP conjugate, resulting in colorimetric signal production (Figure 7a). The simple method of biotin conjugation through duplex formation avoids the need for expensive direct conjugation of functional groups to the termini of aptamers [29].

One interesting feature of our sandwich ELASA is its independency of any blocking steps before target incubation. Even without any blocking steps, low background binding is observed. This could be plausible because the coating of the wells with high concentrations of the negatively charged TDENV-3 aptamer can cover most of the hydrophobic surface. Hence, the surface area available for nonspecific binding is reduced, resulting in a significantly lower background OD reading. As a whole, the developed rapid, blocking-free aptamer-based sandwich assay reduces the overall time taken to complete the assay from more than a day to 4 h. The use of aptamers as both the capture and reporter probes creates a unique sandwich ELASA system that eliminates the partial dependency on target-specific antibodies, as seen with other reported aptamer-based sandwich ELASA that still rely on antibodies [30,31]. The LOD of the developed sandwich ELASA assay is 6.3 nM (Figure 7b).

### 3.13. Stabilized Aptamers Have Improved Binding Rates to the Target NS1

Previous studies have proven that the stabilization of the RNA aptamers using 2′-fluoro substitution can increase their melting temperature, chemical stability and binding affinities without changing the conformation of the aptamers [32,33]. The usage of the stabilized aptamers in improving the performance of the sandwich ELASA assay was investigated. Prior to this, the stabilized TDENV-3 (STDENV-3) aptamer was first analysed in the direct ELASA. The stabilized aptamers were observed to have faster *on*-rate during target binding, as seen from the time course analysis shown in Figure 7c. STDENV-3 and -6a displayed complete target binding within 15 min of incubations, while the unmodified aptamers require a minimum of 1 h in direct ELASA. This is advantageous, as faster target binding can further reduce the time of the sandwich assay. When analysed using direct ELASA, the stabilized TDENV-3 (STDENV-3) aptamer exhibited a similar LOD to the LOD of its un-stabilized version (Figure 7c). In fact, the stabilized TDENV-6a exhibited an improved performance compared to the unmodified TDENV-6a, whereby its LOD can reach down to 12.5 nM with an enhanced or higher OD reading, which is more than 0.6 (Figure 7d). 

### 3.14. Both the Unmodified and Stabilized Aptamers Are Stable in Serum up to 6 h

**Figure 8:**
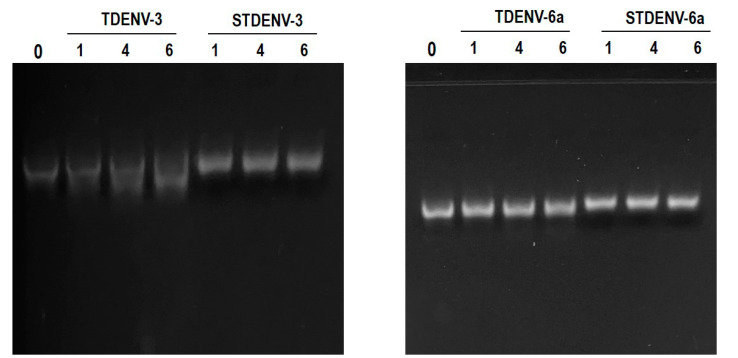
Agarose gel electrophoresis of aliqouts of the reaction mixtures of the TDENV and STDENV aptamers incubated in 1% serum collected after 0, 1, 4 and 6 h.

The stability of both the unmodified (TDENV) and stabilized aptamers (SDENV) was analysed using gel electrophoresis to investigate the extent of any degradation of the aptamers in serum. We observed that both the TDENV and STDENV aptamers were relatively stable with no degradation in 1% serum up to 6 h of incubation (Figure 8). As they are quite resistant to nuclease degradation both in unmodified and stabilized forms, they can be integrated into sandwich assays to be used for the diagnostic detection of NS1 in serum.

### 3.15. Repeated Incubations Improved the Performance of the Sandwich ELASA

**Figure 9:**
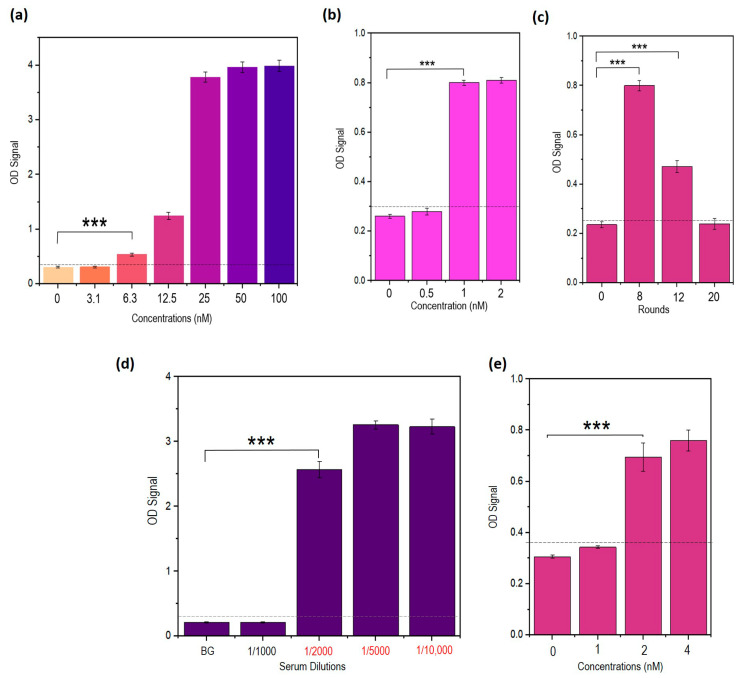
(**a**) Determination of the LOD of sandwich ELASA using STDENV-3 as capture probe and STDENV-6a as reporter probes (*** = *p* < 0.001). (**b**) Determination of the LOD of sandwich ELASA using repetitive incubation of the target NS1 (*** = *p* < 0.001). (**c**) Determination of the numbers of repeated incubations for significant signal production (*** = *p* < 0.001). (**d**) OD readings of the detection of NS1 at a fixed concentration using different dilutions of serum (*** = *p* < 0.001). (**e**) Determination of LOD of sandwich ELASA carried out with repeated incubations of target NS1 in serum diluted at a dilution factor of 1:2000 (*** = *p* < 0.001). The figures shown are representative figures of independent experiments performed in triplicate.

Repeated incubation of the captured probe with the target NS1 was hypothesized to increase the LOD. In this study, the target antigen NS1 was incubated multiple times with the STDENV-3 capture probe. Repeated incubation was speculated to increase the amount of target antigen NS1 bound to the immobilized STDENV aptamers in sandwich ELASA, potentially improving its performance. Due to the enhanced *on*-rates of STDENV-3, complete NS1 binding can be achieved within this short window of target incubation, which can further maximize the target NS1 captured by the capture probe. Furthermore, the performance of the sandwich ELASA using the stabilized aptamers showed an overall improvement in terms of OD reading with the LOD of 6.3 nM (Figure 9a).

As for the repeated incubation, each incubation round was carried out for 15 min with a total of eight times, whereby the concentrations of 2 nM, 1 nM and 0.5 nM of NS1 were tested. Figure 9b shows that the LOD for the assay is 1 nM. As the amount of NS1 protein in serum is reported to be within a range of 10–50 µg/mL during the initial 7-day period of infection, which is equivalent to approximately 185–925 nM, [4,34] this assay can be sensitive enough to reach the clinical range of asymptomatic dengue fever. The number of repeated incubations was also increased up to 20 rounds to increase the LOD of the assay. Based on Figure 9c, the maximum number of incubations that can be carried out to generate a significant OD reading distinguishable from the background reading is 8, while 12 and 20 times resulted in less significant OD readings. The poor OD readings obtained using incubation rounds of more than 8 could be due to the dissociation of the target NS1 bound to the immobilized aptamers that could occur as the time of incubation exceeds the time period of aptamer–target association. As a result, the cumulative amount of the captured NS1 reduces, causing the OD readings to drop. The result suggests that a maximum of eight repeated incubations is sufficient to obtain sensitive signal generation.

### 3.16. Developed Sandwich ELASA Detects Clinical NS1 Levels Indicative of Dengue Infection in Human Serum

To mimic clinical samples, commercial human serums were spiked with recombinant dengue NS1. Serial dilutions of serum in PBS buffers were made; 1:100 (1%), 1:1000 (0.1%), 1:2000 (0.05%), 1:5000 (0.02%) and 1:10,000 (0.01%), and they were spiked with 100 nM of NS1 each to determine the dilution factor at which the optimum aptamer–target binding occurs. Figure 9d reveals that the dilution of 1:2000 (0.05%) is the minimum dilution of serum that can be used for a significant OD reading. The degree of serum interferences was much higher at lower serum dilutions (1:1000). The LOD of the developed sandwich assay using the stabilized aptamers (STDENV-3 and STDENV-6a) was further analysed with the serum dilution (1:2000) utilizing the repeated target incubation strategy. A LOD of 2 nM can be achieved (Figure 9e). The LOD of the developed sandwich ELASA falls within the range of NS1 concentration indicative of dengue infection [4,34].

Contemporary NS1 detection assay comprises only antibody-based recognition elements. Antibodies are expensive and demonstrate significant batch-to-batch variation. In this study, the developed diagnostic ELISA platform consists of aptamer-based recognition elements. This translates to a significantly lower production cost compared to antibody-based assays, across both the selection procedures and their diagnostic implementations. The diagnostic platform, the sandwich assay specifically, reaches the required clinical range of dengue NS1 levels in patient serum, suggesting their high potentiality in a clinical setting.

## 4. Conclusions

In this study, we isolated DENV-3 and DENV-6, with the dissociation constant values estimated at 37.57 ± 10.34 nM and 41.40 ± 9.29 nM, respectively. These aptamers can be further truncated to TDENV-3 and TDENV-6a. These truncated aptamers are highly specific against the target dengue NS1 with no cross-reactivity demonstrated against the NS1 of the Zika virus, the E2 of the Chikungunya virus or the LipL32 protein of Leptospira. The aptamer-based sandwich ELASA assay for the detection of dengue NS1 was successfully developed, achieving a LOD of 2 nM in human serum diluted at the dilution factor of 1:2000.

## Figures and Tables

**Table 1:** The parameters used for eleven SELEX cycles. Nitrocellulose filter membrane-based partitioning was used for all the SELEX cycles except for the cycles with the notations ‘*’, in which ELISA microtiter plate-based partitioning was used.

SELEX Cycle	RNA Pool (µM)	Competitor (µM)	NS1 Protein (µM)	PCR Amplification Cycles
1	6.6	10.2	2.5	8
2	3.9	30.6	2.0	8
3 *	2.1	50.9	1.5	8
4	1.6	81.6	1.0	8
5 *	1.1	112.2	0.75	8
6	0.8	152.9	0.5	8
7	0.4	193.7	0.25	6
8 *	0.3	203.9	0.125	6
9	0.3	203.9	0.125	6
10	0.2	203.9	0.1	5
11 *	0.1	203.9	0.05	8

**Table 2:** RNA aptamer candidate sequences and their percentage of appearance (%).

Class	Sequence	Number of Identical Sequences	Percentage of Appearance (%)
**DENV-1**	GGAGCUCAGCCUUCACUGCUUAGUCCCGUCAACGUCGGAGGUGACAGCCGUUGGCGGGCCGGCUUGGCGUUAACAUGAAGGCACCACGGUCGGAUCCAC	5	17.86
**DENV-2**	GGAGCUCAGCCUUCACUGCUGUCAGAUAACAUGCAUGAUAGACUGAUGAUCGUCCAUGUUUGAAACUGAUCAGUAUCGAGGCACCACGGUCGGAUCCAC	3	10.71
**DENV-3**	GGAGCUCAGCCUUCACUGCUUUGAUCUCGUGGGGGUGUGUCGCGGGAGACACCAUGGAAUAUAUGGCUGAUUUCAUGUGGGCACCACGGUCGGAUCCAC	5	17.86
**DENV-4**	GGAGCUCAGCCUUCACUGCGUGAUGCGUAGCUCGAUACAAUGGUUGUCUUAAAAGUGACGCCUUUGUCGAAAACGAGAUGGCACCACGGUCGGAUCCAC	5	17.86
**DENV-5**	GGAGCUCAGCCUUCACUGCUUUAGGUGCCCAUAGGGACGGGUUGGGAUCAUAGUUCGUGAAGGUGCUAUGAUACGGGAGGGCACCACGGUCGGAUCCAC	5	17.86
**DENV-6**	GGAGCUCAGCCUUCACUGCCGUAGUCGUAUCUCCAUUACCCAGAAUGGUGAUGCGCCGUGAAGAGCGGUUUAGGGAAUUGGCACCACGGUCGGAUCCAC	3	10.71
**DENV-7**	GGAGCUCAGCCUUCACUGCUUGCUUUUGGGUGCCGUACGCAUUGCGGCAGGGGGAAGAGGAGGGUAGCGACCAGUCGAAGGCACCACGGUCGGAUCCAC	2	7.14

**Table 3:** Z-score values computed for the docking of TDENV and DENV with target NS1 dengue using different docking softwares.

**TDENV**	**Haddock Z-Score**	**Autodock Z-Score**	**Patchdock Z-Score**	**Total Z-Score**
**3**	−1.2	−2.37	−1.77	−5.34
**4**	−1.3	−1.29	−1.66	−4.25
**6a**	−2.2	−1.34	−1.72	−5.26
**6b**	−1.5	−1.45	−1.82	−4.77
**DENV**	**Haddock Z-score**	**Autodock Z-score**	**Patchdock Z-score**	**Total Z-score**
**3**	−1.3	−1.40	−1.54	−4.24
**4**	−1.3	−2.10	−2.04	−5.44
**6**	−1.6	−1.38	−1.85	−4.83

**Table 4:** Z-score values computed for control sequences.

No.	Control Sequences	Haddock Z-Score	Autodock Z-Score	Patchdock Z-Score	Total Z-Score
**C1**	AAUACAAAUUGUGUU	−2.3	−2.00	−0.30	−4.60
**C2**	CAUAGCAGACAGCUAUC	−2.5	−1.80	−0.50	−4.80
**C3**	AGUAAUACUCGCUGC	−1.6	−1.90	−1.00	−4.50

**Table 5:** Z-score values computed for dual docking interactions.

Docked Models	Haddock Z-Score	Patchdock Z-Score
**TDENV-3/NS1—TDENV-6a**	−2.3	−1.76
**TDENV-6a/NS1—TDENV-3**	−1.6	−1.57

## Data Availability

The data presented in this study are available on request from the corresponding author.

## Supplementary Materials

The following supporting information can be downloaded at: https://www.mdpi.com/article/10.3390/biology12050722/s1, Supplementary Table S1: List of primers and templates for the preparation of truncated aptamers.
