# Supplementary material for: The Diagnostic Potential of RNA Aptamers against the NS1 Protein of Dengue Virus Serotype 2
Source: Biology (Basel). 2023 May 15;12(5):722. doi: 10.3390/biology12050722 (PMC10215423; doi:10.3390/biology12050722)
Supplement: Supplementary file 1 [file biology-12-00722-s001.zip › biology-2305658-supplementary.pdf]

Supplementary Table S1: List of primers and templates for the preparation of truncated aptamers

**NAME**	**SEQUENCE (5′ – 3′)**
**TDENV-3**	
Forward Primer	TCTAATACGACTCACTATAGGCCTTCACTGCTTTGAT
Reverse Primer	TTTTTTTTTTTTTTTTTTTTTTTTGGCCATATATTCCATG
Template	GGCCTTCACTGCTTTGATCTCGTGGGGGTGTGTCGCGGGAGA CACCATGGAATATATGGCC
**TDENV-6a**	
Forward Primer	TCTAATACGACTCACTATAGCCGTAGTCGTATCTCC
Reverse Primer	TTTTTTTTTTTTTTTTTTTTTTTTCGGCGCATCACCATTCTGGG
Template	TCTAATACGACTCACTATAGCCGTAGTCGTATCTCCATTACC CAGAATGGTGATGCGCCG
**TDENV-6b**	
**Forward Primer**	TCTAATACGACTCACTATAGAGCTCAGCCTTCACTGCCG
**Reverse Primer**	TTTTTTTTTTTTTTTTTTTTTTTTCCCTAAACCGCTCTTCACGGCGCA
**Template**	GAGCTCAGCCTTCACTGCCGTAGTCGTATCTCCATTACCCA GAATGGTGATGCGCCGTGAAGAGCGGTTTAGGG
